# Validity and Reproducibility of the Peer Assessment Rating Index Scored on Digital Models Using a Software Compared with Traditional Manual Scoring

**DOI:** 10.3390/jcm10081646

**Published:** 2021-04-13

**Authors:** Arwa Gera, Shadi Gera, Michel Dalstra, Paolo M. Cattaneo, Marie A. Cornelis

**Affiliations:** 1 Section of Orthodontics, Department of Dentistry and Oral Health, Aarhus University, C 8000 Aarhus, Denmark; arwa@dent.au.dk (A.G.); shadigera@dent.au.dk (S.G.); michel.dalstra@dent.au.dk (M.D.); 2 Faculty of Medicine, Dentistry and Health Sciences, Melbourne Dental School, University of Melbourne, Carlton, VIC 3053, Australia; paolo.cattaneo@unimelb.edu.au

**Keywords:** orthodontics, CAD/CAM, PAR index, dental models, digital models, clinical

## Abstract

The aim of this study was to assess the validity and reproducibility of digital scoring of the Peer Assessment Rating (PAR) index and its components using a software, compared with conventional manual scoring on printed model equivalents. The PAR index was scored on 15 cases at pre- and post-treatment stages by two operators using two methods: first, digitally, on direct digital models using Ortho Analyzer software; and second, manually, on printed model equivalents using a digital caliper. All measurements were repeated at a one-week interval. Paired sample *t*-tests were used to compare PAR scores and its components between both methods and raters. Intra-class correlation coefficients (ICC) were used to compute intra- and inter-rater reproducibility. The error of the method was calculated. The agreement between both methods was analyzed using Bland-Altman plots. There were no significant differences in the mean PAR scores between both methods and both raters. ICC for intra- and inter-rater reproducibility was excellent (≥0.95). All error-of-the-method values were smaller than the associated minimum standard deviation. Bland-Altman plots confirmed the validity of the measurements. PAR scoring on digital models showed excellent validity and reproducibility compared with manual scoring on printed model equivalents by means of a digital caliper.

## 1. Introduction

For high standards of orthodontic treatment quality to be maintained, frequent monitoring of treatment outcomes is a prerequisite for orthodontists. The orthodontic indices widely used in clinical and epidemiological studies to evaluate malocclusion and treatment outcome [1,2,3] include the Index of Orthodontic Treatment Need (IOTN) [4], the Index of Complexity Outcome and Need (ICON) [5], the American Board of Orthodontics objective grading system (ABO-OGS) index [6], the Peer Assessment Rating (PAR) index [7], and the Dental Aesthetic Index (DAI) [8].

The PAR [7] is an occlusal index developed to provide an objective and standardized measure of static occlusion at any stage of treatment using dental models. Therefore, this index is widely used among clinicians whether it is in the private or the public sector, including educational institutions. In fact, in the UK, the use of this index is obligatory in all orthodontic clinics offering public service to audit orthodontic treatment outcome, and it is used as a measure for quality assurance. The assessment of malocclusion can be recorded at any stage of orthodontic treatment, such as pre- and/or post-treatment, whereas the difference in PAR scores between two stages evaluates treatment outcome. Its validity and reliability on plaster models have been reported in England [9], as well as in the United States [10]. It is also a valid tool for measuring treatment need [11].

The use of plaster models and digital calipers has been acknowledged as the gold standard for study model analyses and measurements [12,13]. Traditionally, PAR scoring is performed on plaster models by means of a PAR ruler or a combination of a digital caliper and a conventional ruler. Several studies have used this method to assess malocclusion, treatment need, treatment outcomes, and stability of occlusion [11,14,15,16,17,18,19]. However, the human-machine interface has evolved, influencing orthodontics significantly, shifting from a traditional clinical workflow towards a complete digital flow, where digital models have become more prevalent. Digital models enable patients’ records to be stored digitally and for essential orthodontic assessments, such as diagnosis, treatment planning, and assessment of treatment outcome, to be carried out virtually through several built-in features, such as linear measurements [13,20], Bolton analysis, space analyses [21], treatment planning [22], and PAR scoring [12,23]. However, this modern paradigm demands adaptation and assessment of applicability in orthodontic clinical work. Nevertheless, assessments of the validity and reproducibility of 3-dimensional (3-D) digital measurement tools remain scarce.

Digital models can be obtained either directly or indirectly and can be printed or viewed on a computer display. Scanned-in plaster models are the indirect source of digital models and are as valid and reliable as conventional plaster models [12,13,24,25]. In the present study, digital models were obtained directly from an intraoral scanner. Emphasis on evaluating a complete virtual workflow was recently implemented by three studies [26,27,28]. Brown et al. [26] concluded that 3-D printed models acquired directly from intraoral scans provided clinically acceptable models and should be considered as a viable option for clinical applications. Luqmani et al. [28] assessed the validity of digital PAR scoring by comparing manual PAR scoring using conventional models and a PAR ruler with automated digital scoring for both scanned-in models and intraoral scanning (indirect and direct digital models, respectively). The authors concluded that automated digital PAR scoring was valid and that there were no significant differences between direct and indirect digital model scores.

However, to our knowledge, the digital non-automated PAR index scoring tool of the Ortho Analyzer software has not been previously validated. Therefore, the purpose of this study was to assess the validity and reproducibility of digital scoring of the PAR index and its components on digital models using this software, compared with conventional manual scoring on printed model equivalents.

## 2. Materials and Methods

### 2.1. Sample Size Calculation

A sample size calculation was performed using the formula given by Walter et al. [29]. For a minimum acceptable reliability (intra-class correlation (ICC)) of 0.80, an expected reliability of 0.96, with a power of 80% and a significance of 0.05, a sample of 12 subjects was needed. It was decided to extend the sample to 15 subjects.

### 2.2. Setting

The study was conducted at the Section of Orthodontics, School of Dentistry and Oral Health, Aarhus University, Denmark. This type of study is exempt from ethics approval in Denmark (Health Research Ethics Committee-Central Jutland, Denmark, case no. 1-10-72-1-20).

### 2.3. Sample Collection

The study sample consisted of 15 consecutive patient records (the first record being randomly chosen) selected from the archives, according to the following inclusion criteria: (1) patients had undergone orthodontic treatment with full fixed appliances at the postgraduate orthodontic clinic between 2016 and 2018; and (2) digital models before and after treatment were available. No restrictions were applied with regards to age, initial malocclusion and end-of-treatment results.

The digital models for both treatment stages; pre-treatment (T0) and post-treatment (T1), had been directly generated by a TRIOS intraoral scanner (3Shape, Copenhagen, Denmark) as stereolithographic (STL) files, imported and analyzed through Ortho Analyzer software (3Shape, Copenhagen, Denmark). Subsequently, 30 digital models were printed, to generate 15 model equivalents for each stage, by means of model design software (Objet Studio, Stratasys, Eden Prairie, MN, USA) and a 3-D printing machine (Polyjet prototyping technique; Objet30 Dental prime, Stratasys, Eden Prairie, MN, USA), in the same laboratory and with the same technique.

### 2.4. Measurements

**Figure 1:**
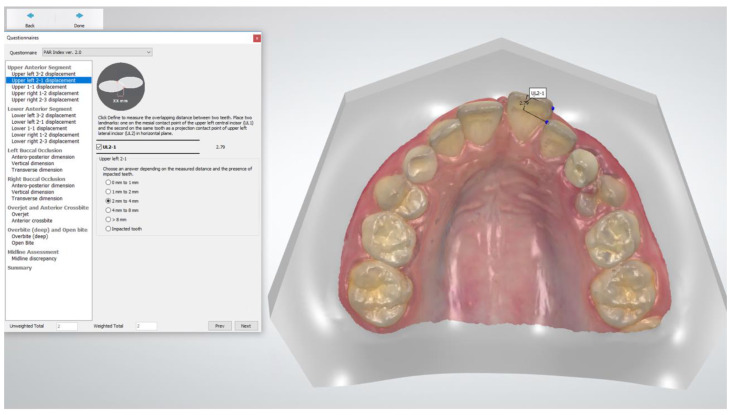
Peer Assessment Rating (PAR) index scoring using Ortho Analyzer software. Anterior component scoring contact point displacement between teeth 21 and 22.

The PAR scoring was performed at T0 and T1 by two methods: (1) digitally, on the direct digital models using a built-in feature of the Ortho Analyzer software (Figure 1); and (2) manually, on the printed model equivalents using a digital caliper (Orthopli, Philadelphia, PA, USA), measured to the nearest 0.01 mm with an orthodontic tip accuracy of 0.001, except for overjet and overbite, which were measured with a conventional ruler. Two operators (AG and SG), previously trained and calibrated in the use of both techniques, performed all the measurements independently. Reproducibility was determined by repeated measurements on all models by both methods and by both raters at a one-week interval and under identical circumstances.

The PAR scoring was performed using the UK weighting system according to Richmond et al. [7] and included five components, scoring various occlusal traits which constitute malocclusion: anterior segment, posterior segment, overjet, overbite, and centerline (Table 1). The scores of the traits were summed and multiplied by their weight. The component-weighted PAR scores were summed to constitute the total weighted PAR score. Essential information about each case was considered, such as impacted teeth, missing or extracted teeth, plans for any prosthetic replacements, and restorative work previously carried out that affected the malocclusion.

### 2.5. Statistical Analyses

Data collection and management were performed by means of the Research Electronic Data Capture (REDCap) tool hosted at Aarhus University [30,31]. Statistical analyses were carried out with Stata software (Release 16, StataCorp. 2019, College Station, TX, USA).

Descriptive statistics were used to analyze the total PAR scores at different time points, between raters and methods used. Paired sample t-tests were used to compare PAR scoring between both methods and raters at a significance level of <0.05. Both methods were assessed by ICC for intra- and inter-rater reproducibility. Intra- and inter-rater variability were determined by calculation of the error of the method according to Dahlberg’s formula [32]. The agreement between the digital and manual scoring methods performed by the two raters was determined by a scatter plot and Bland-Altman plots.

## 3. Results

### 3.1. Validity

**Figure 2:**
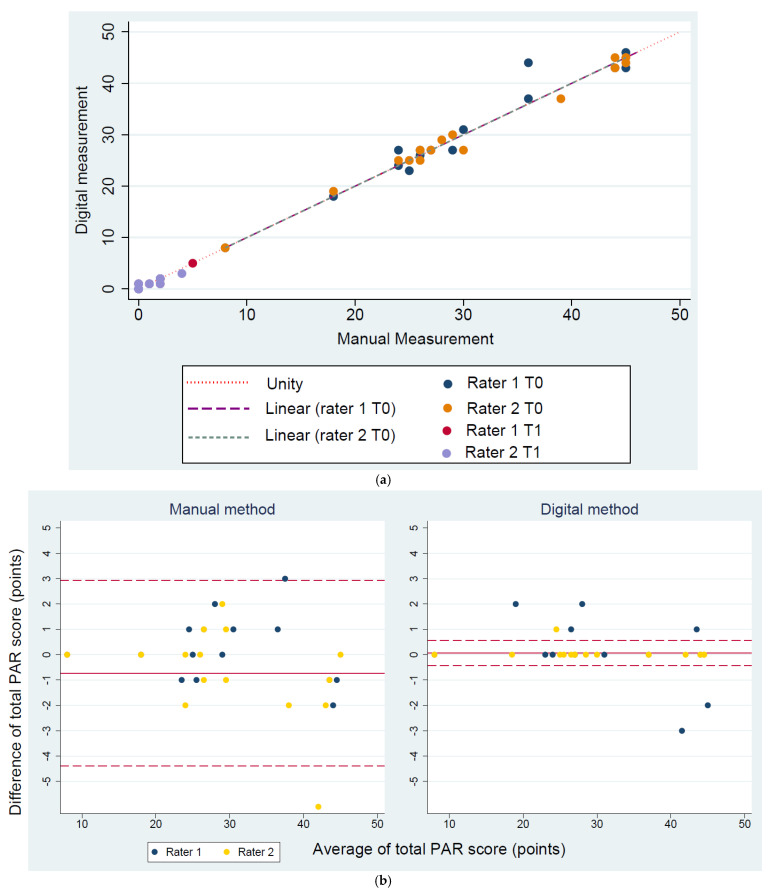
(**a**) Scatter plot of the total weighted PAR scores measured by both digital and manual methods and both raters, with a line of unity; (**b**) Bland-Altman plots: inter-rater agreement for the total PAR scores measured by the digital and manual PAR scoring methods at T0.

Paired-sample t-tests showed no significant differences in the mean total PAR scores and in the PAR components between both methods (Table 2 and Table 3). The scatter plot (Figure 2a) and Bland-Altman plots (Figure 2b) illustrate agreement of the measurements conducted with both methods.

### 3.2. Reproducibility

ICC for the total PAR scores and the PAR components at both time points and for both methods fell in the 0.95–1.00 range for intra- and inter-rater reproducibility (Table 2 and Table 3). All error-of-the-method values for the total PAR score and its components were smaller than the associated minimum standard deviation.

## 4. Discussion

Orthodontic model analysis is a prerequisite for diagnosis, evaluation of treatment need, treatment planning and analysis of treatment outcome. The present study assessed the validity and reproducibility of PAR index scoring for digital models and their printed model equivalents.

Digitization of plaster models (scanned-in) was introduced in the 1990s [33]. The advantages of digital models include the absence of physical storage requirements, instant accessibility, and no risk of breakage or wear [22,34]. The analysis of scanned-in models is as valid as that for plaster models [24]. However, over the last ten years, technology has evolved drastically, offering high image resolution of digital models and upgraded platforms needed for their analyses. Hence, intraoral scanning has gained popularity worldwide. Several studies have confirmed the accuracy of direct digital models to be as accurate as that of plaster models. Consequently, direct digital models are used as an alternative to conventional impression techniques and materials [35,36]. In the present study, we used the direct digital model technique.

Numerous intraoral scanners and software were developed over the last decade, with various diagnostic tools. In the present study, Ortho Analyzer software was used for scoring the digital models, and a digital caliper was used for scoring the printed models. Analyzing digital models can be associated with some concerns. The main concern with the use of digital software is, in fact, adjusting the visualization of a 3-D object on a two-dimensional screen. An appropriate evaluation requires a correct model orientation. For instance, in this study, cross bites were difficult to visualize, and rotation of the model was required to fully comprehend the magnitude of the cross bite. This problem was also reported by Stevens et al. [12]. In addition, segmentation of the dental crowns, which is an inevitable step to create a virtual setup before carrying out the scoring, is time-consuming. To ensure accurate tooth displacement measures, one should pay attention when placing the points, parallel to the occlusal plane, rotating the model adequately to facilitate good visualization of the contact points.

Another concern when dental measurements are performed on printed models, is the consideration of the printing technique and the model base design. Two studies [26,27] evaluated the accuracy of printed models acquired from intraoral scans. Brown et al. [26] compared plaster models with printed models using two types of 3-D printing techniques and concluded that both digital light processing (DLP) and polyjet printers produced clinically acceptable models. Camardella et al. [27] compared 3-D printed models with different base designs using two types of printing techniques and concluded that 3-D printed models from intraoral scans created with the polyjet printing technique were accurate, regardless of the model base design. By contrast, 3-D printed models with a horseshoe-shaped base design printed with a stereolithography printer showed a significant transversal (intra-arch distances) contraction, and a horseshoe-shaped base with a posterior connection bar was accurate compared with printed models with a regular base. Therefore, in the present study, a polyjet printing technique and regular model base designs were used to ensure accuracy.

Luqmani et al. [28] compared automated PAR scoring of direct and indirect digital models (CS 3600 software; Carestream Dental, Stuttgart, Germany) with manual scoring of plaster models using the PAR ruler. The authors found that manual PAR scoring was the most time-efficient, whereas indirect digital model scoring was the least time-efficient. The latter had minor dental cast faults that led to time-consuming software adjustments. However, automated scoring was more efficient than the software scoring used by Mayers et al. [23], which required operators to identify each relevant landmark. Hence, indirect scoring depends on the quality of the dental casts and can be time-consuming, depending on the software used. In the present study, PAR scoring was not possible as the software used (Ortho Analyzer) does not have the feature of automated scoring.

In the present study, the slightly higher reproducibility of PAR scoring for the digital method was not significant, with both methods proving to be highly reproducible. The high reproducibility of the manual method coincides with the results of Richmond et al. [7]. The reproducibility and variability of the direct digital method were similar to the findings described by Luqmani et al. [28]. Furthermore, the limited variability between both methods demonstrated the high validity of the digital method compared with the conventional manual method, used as the gold standard.

## 5. Conclusions

PAR scoring on digital models using a software showed excellent reproducibility and presented good validity compared with manual scoring, considered as the gold standard.

## Figures and Tables

**Table 1:** The PAR index components and scoring.

	PAR Component	Assessment	Scoring	Weighting
1	Anterior ^†^	Contact point displacement	0–4	× 1
		Impacted incisors/canines	5	
2	Posterior ^‡^	Sagittal occlusion	0–2	× 1
		Vertical occlusion	0–1	
		Transverse occlusion	0–4	
3	Overjet ^§^	Overjet	0–4	× 6
		Anterior crossbite	0–4	
4	Overbite ^¶^	Overbite	0–3	× 2
		Open bite	0–4	
5	Centerline	Deviation from dental midline	0–2	× 4
	Total		Unweighted PAR score	Weighted PAR score

^†^ Measured from the mesial contact point of canine on one side to the mesial contact point of canine on the opposite side (upper and lower arches) and recorded in millimeters as the shortest distance between contact points of adjacent teeth and parallel to the occlusal plane. ^‡^ Measured from the distal contact point of canine to the mesial contact point of permanent molar or last molar (right and left sides). ^§^ Measured as the largest horizontal distance parallel to the occlusal plane from the labial incisor edge of the most prominent upper incisor to the labial surface of the corresponding lower incisor. ^¶^ Recorded as the largest vertical overlap or open bite between upper incisors and lower incisors.

**Table 2:** Intra-rater variability (error of the method and Minimum Standard Deviation (MSD)) and reproducibility (ICC) according to time point and method, for both raters. Paired sample *t*-tests comparing both methods.

			Rater I							Rater II						
PAR Index Scoring and Timepoint		Scoring Method	Mean (SD) ^a^	Err. Method *^,a^	MSD ^a^	ICC	[95% CI]		*p*-Value (Paired *t*-Test)	Mean (SD) ^a^	Err. Method *^,a^	MSD ^a^	ICC	[95% CI]		*p*-Value (Paired *t*-Test)
T0	Total PAR	Manual	29.5 (10.1)	0.9	10.1	0.99	0.99	1.00	0.16	30.5 (10.7)	1.4	10.1	0.99	0.98	1.00	0.74
		Digital	30.2 (10.5)	0.9	10.1	1.00	0.99	1.00		30.4 (10.4)	0.6	10.3	1.00	0.99	1.00	
T1		Manual	1.1 (1.4)	0.2	1.4	0.99	0.99	1.00	0.72	1.2 (1.3)	0.4	1.2	0.95	0.84	0.98	0.49
		Digital	1.2 (1.3)	0.2	1.1	0.98	0.94	0.99		1.0 (0.9)	0.2	1.0	0.98	0.95	0.99	
T0	Lower anterior	Manual	4.1 (4.0)	0.5	4.0	0.99	0.99	1.00	0.12	4.4 (4.2)	0.6	4.1	0.99	0.97	1.00	0.60
		Digital	4.4 (3.6)	0.2	3.5	1.00	0.99	1.00		4.5 (3.7)	0.4	3.7	0.99	0.98	1.00	
T1		Manual	0.3 (0.6)	0.0	0.6	1.00	-	-	0.16	0.3 (0.6)	0.0	0.6	1.00	-	-	0.16
		Digital	0.1 (0.3)	0.0	0.4	1.00	-	-		0.1 (0.3)	0.0	0.4	1.00	-	-	
T0	Upper anterior	Manual	6.1 (2.8)	0.6	2.6	0.97	0.92	0.99	0.45	6.3 (2.8)	0.7	2.6	0.98	0.93	0.99	0.50
		Digital	6.3 (2.8)	0.7	2.6	0.96	0.89	0.99		6.4 (2.7)	0.6	2.6	0.98	0.95	0.99	
T1		Manual	0.2 (0.4)	0.0	0.4	1.00	-	-	0.19	0.2 (0.4)	0.0	0.4	1.00	-	-	0.19
		Digital	0.3 (0.5)	0.1	0.5	1.00	-	-		0.3 (0.5)	0.1	0.5	1.00	-	-	
T0	Posterior	Manual	1.3 (1.5)	0.4	1.4	0.97	0.91	0.99	0.16	1.4 (1.5)	0.0	1.5	1.00	-	-	0.33
		Digital	1.5 (1.6)	0.0	1.6	1.00	-	-		1.5 (1.6)	0.0	1.6	1.00	-	-	
T1		Manual	0.2 (0.6)	0.2	0.6	0.95	0.84	0.98	0.67	0.2 (0.6)	0.0	0.6	1.00	-	-	-
		Digital	0.3 (0.6)	0.0	1.5	1.00	-	-		0.3 (0.6)	0.0	0.6	1.00	-	-	
T0	Overjet	Manual	12.0 (7.5)	0.0	7.5	1.00	-	-	-	12.0 (7.5)	0.0	7.5	1.00	-	-	-
		Digital	12.0 (7.5)	0.0	7.5	1.00	-	-		12.0 (7.5)	0.0	7.5	1.00	-	-	
T1		Manual	0.0 (0.0)	0.0	0.0	** -	-	-	-	0.0 (0.0)	0.0	0.0	-	-	-	-
		Digital	0.0 (0.0)	0.0	0.0	** -	-	-		0.0 (0.0)	0.0	0.0	-	-	-	
T0	Overbite	Manual	3.1 (1.8)	0.0	1.8	1.00	-	-	0.33	2.9 (1.9)	0.5	1.8	0.96	0.89	0.99	1.00
		Digital	3.0 (1.8)	0.4	1.8	0.98	0.94	0.99		2.9 (1.8)	0.0	1.8	1.00	-	-	
T1		Manual	0.3 (0.7)	0.5	0.7	1.00	-	-	-	0.3 (0.7)	0.0	0.7	1.00	-	-	-
		Digital	0.3 (0.7)	0.5	0.7	1.00	-	-		0.3 (0.7)	0.4	0.5	1.00	-	-	
T0	Centerline	Manual	2.1 (3.0)	0.0	3.0	1.00	1.00	1.00	0.33	2.1 (3.0)	0.0	3.0	1.00	1.00	1.00	0.33
		Digital	2.0 (2.7)	0.8	2.6	0.96	0.89	0.99		1.9 (2.6)	0.0	2.6	1.00	-	-	
T1		Manual	0.0 (0.0)	0.0	0.0	** -	-		-	0.0 (0.0)	0.0	0.0	-	-	-	-
		Digital	0.0 (0.0)	0.0	0.0	** -		-		0.0 (0.0)	0.0	0.0	-	-	-	

* Error of the method according to Dahlberg’s formula. ** ICC could not be calculated as all the scores for overjet and centerline were = 0. ^a^ Expressed in score points unit.

**Table 3:** Inter-rater variability (error of the method and Minimum Standard Deviation (MSD)) (using the second measurements) and reproducibility (ICC) according to time point and method. Paired sample *t*-tests comparing both raters.

PAR Index Scoring and Timepoint		Scoring Method	Rater I	Rater II	Rater I vs. II					
			Mean (SD) ^a^	Mean (SD) ^a^	Err. Method ^a,^*	MSD ^a^	ICC	[95% CI]		*p*-Value (Paired *t*-Test
T0	Total PAR	Manual	29.6 (10.0)	30.3 (9.72)	1.8	10.3	0.99	0.99	1.00	0.20
		Digital	30.2 (10.2)	30.4 (10.2)	0.8	10.6	1.00	0.99	1.00	0.55
T1		Manual	1.1 (1.4)	1.1 (1.4)	0.2	1.2	0.99	0.96	0.99	1.00
		Digital	1.1 (1.2)	1.0 (1.0)	0.4	1.0	0.98	0.95	0.99	0.26
T0	Lower anterior	Manual	4.1 (4.0)	4.5 (4.4)	0.6	4.0	0.99	0.98	1.00	0.06
		Digital	4.4 (3.6)	4.5 (3.7)	0.7	3.5	0.99	0.96	0.99	0.64
T1		Manual	0.3 (0.6)	0.3 (0.4)	0.0	0.6	1.00	-	-	-
		Digital	0.0 (0.1)	0.1 (0.2)	0.0	0.3	1.00	-	-	-
T0	Upper anterior	Manual	6.1 (2.8)	6.3 (2.8)	0.5	2.8	0.99	0.97	1.00	0.33
		Digital	6.4 (2.8)	6.4 (2.7)	0.6	2.7	0.99	0.98	1.00	0.38
T1		Manual	0.1 (0.3)	0.1 (0.2)	0.3	0.3	1.00	0.49	0.94	-
		Digital	0.4 (0.7)	0.1 (0.3)	0.6	0.3	1.00	0.64	0.96	-
T0	Posterior	Manual	1.3 (1.4)	1.4 (1.5)	0.4	1.4	0.98	0.93	0.99	0.58
		Digital	1.5 (1.6)	1.5 (1.6)	0.0	1.6	1.00	-	-	-
T1		Manual	0.2 (0.6)	0.2 (0.6)	0.0	0.6	0.99	0.96	0.99	0.33
		Digital	0.3 (0.6)	0.2 (0.6)	0.2	0.6	0.95	0.84	0.98	0.33
T0	Overjet	Manual	12 (7.5)	12 (7.5)	0.0	7.5	1.00	-	-	-
		Digital	12 (7.5)	12 (7.5)	0.0	7.5	1.00	-	-	-
T1		Manual	0.0 (0.0)	0.0 (0.0)	0.0	0.0	** -	-	-	-
		Digital	0.0 (0.0)	0.0 (0.0)	0.0	0.0	** -	-	-	-
T0	Overbite	Manual	3.1 (1.8)	3.1 (1.8)	0.4	1.8	0.99	0.97	1.00	0.16
		Digital	3.1 (1.8)	2.9 (1.8)	0.4	1.8	0.97	0.92	0.99	0.67
T1		Manual	0.3 (0.7)	0.3 (0.7)	0.0	0.5	1.00	-	-	-
		Digital	0.3 (0.7)	0.3 (0.7)	0.0	0.5	1.00	-	-	-
T0	Centerline	Manual	2.1 (3.0)	2.1 (3.0)	0.0	3.0	1.00	-	-	-
		Digital	2.1 (3.0)	1.8 (2.5)	0.8	2.6	0.99	0.97	1.00	0.33
T1		Manual	0.0 (0.0)	0.0 (0.0)	0.0	0.0	** -	-	-	-
		Digital	0.0 (0.0)	0.0 (0.0)	0.0	0.0	** -	-	-	-

* Error of the method according to Dahlberg’s formula, using the second measurements. ** ICC could not be calculated as all the scores for overjet and centerline were = 0. ^a^ Expressed in score points unit.

## Data Availability

The data presented in this study are available on request from the corresponding author.
